# A Low-Temperature Heat Output Photoactive Material-Based High-Performance Thermal Energy Storage Closed System

**DOI:** 10.3390/ma14061434

**Published:** 2021-03-16

**Authors:** Xiangyu Yang, Shijie Li, Jin Zhang, Xiaomin Wang, Yongzhen Wang, Jianguo Zhao

**Affiliations:** 1 College of Materials Science and Engineering, Taiyuan University of Technology, Yingze West Street, Taiyuan 030024, China; yangxiangyu0039@link.tyut.edu.cn (X.Y.); wangxm62@126.com (X.W.); 2 Institute of Carbon Materials Science, Shanxi Datong University, Xingyun Street, Datong 037009, China; li841974@sina.com (S.L.); zhangjin50@hrbeu.edu.cn (J.Z.)

**Keywords:** photothermal conversion material, outstanding heat storage density, long-term storage, low temperature energy output, closed system

## Abstract

Designing and synthesizing photothermal conversion materials with better storage capacity, long-term stability as well as low temperature energy output capability is still a huge challenge in the area of photothermal storage. In this work, we report a brand new photothermal conversion material obtained by attaching trifluoromethylated azobenzene (Azo_F_) to reduced graphene oxide (rGO). Azo_F_-rGO exhibits outstanding heat storage density and power density up to 386.1 kJ·kg^−1^ and 890.6 W·kg^−1^, respectively, with a long half-life (87.7 h) because of the H-bonds based on high attachment density. Azo_F_-rGO also exhibits excellent cycling stability and is equipped with low-temperature energy output capability, which achieves the reversible cycle of photothermal conversion within a closed system. This novel Azo_F_-rGO complex, which on the one hand exhibits remarkable energy storage performance as well as excellent storage life span, and on the other hand is equipped with the ability to release heat at low temperatures, shows broad prospects in the practical application of actual photothermal storage.

## 1. Introduction

With the fast development of society, people’s demand for energy is increasing and the energy issue has now become one of the major problems that human beings need to deal with [1]. Solar energy has the advantages of sufficient reserves, no pollution and economical availability. Efficiently converting and storing solar energy has become an important way to overcome the current energy shortage crisis [2,3,4,5]. Recently, photothermal conversion materials have attracted extensive attention as a new method for storing solar energy [6]. Photothermal conversion materials can store solar energy in chemical bonds through photo-isomerization of units and then releasing the stored energy as thermal energy when exposed to different external stimulus, achieving photothermal conversion within a closed system. Such materials are able to effectively convert light energy into its own chemical bonds and release its stored energy while avoiding the emission of additional greenhouse gases, with the potential to achieve low-cost and large-scale industrial solar storage [7]. However, photothermal conversion materials still have the shortcomings of short storage time, low energy density and inability to achieve energy release under low temperatures, which are key factors limiting its practical application in solar thermal energy storage [8,9].

Owing to its special photoisomerization ability, good structural stability and controllable configuration recoverability, azobenzene and its derivatives with numerous applications [10,11] has received extensive research interest as a kind of photothermal conversion material [12,13]. However, due to the disadvantages of poor storage performance and storage half-life (*τ*_1*/*2_) arising from low isomerization enthalpy (*ΔH*), azobenzene did not exert its full potential in terms of photothermal conversion and storage [14]. To override the above hurdles, great efforts have been made on the basis of molecular design by introducing different substituents and increasing the interaction between molecules [15,16,17]. Grossman et al. [18] reported azobenzene derivatives with bulky aromatic groups as photoactive chemical heat storage materials. Owing to the introduction of bulky phenyl groups, the solid-state azobenzene derivatives not only improve the energy density but also improve the corresponding thermal stability. Bléger et al. [19] reported *o*-Fluoroazobenzenes and derivatives which exhibit an unprecedented long half-life owing to the ortho-fluorine substituent which reduces electron density around the –N=N– double bond. Despite great efforts having been made, it is still an intractable problem to apply azobenzene photothermal conversion material to practical energy storage.

Different from freely dispersed azobenzene, many azobenzene carbon materials were formed by introducing azobenzene into high-strength carbon nanomaterials forms many azobenzene carbon nanocomposites [7,20,21] accompanied by a more closely ordered structure, which have excellent storage capacity and life cycle. The templated, structure modified azobenzene enhance the intermolecular interactions while obtaining a more stable and tightly ordered structure, which jointly improved the storage capacity of azobenzene carbon materials [22,23]. In addition, because of the unique 2D structure and broad surface of graphene with numerous applications [24,25] which contributes to high attachment density, the templated azobenzene/graphene nanomaterials show broad prospects in photothermal storage [26]. Unfortunately, azobenzene carbon nanomaterials still have problems such as difficulty in releasing storage heat at low temperatures and the inability to balance energy density and half-life, which limits their further practical application [27,28]. Therefore, how to simultaneously achieve the improvement of storage capacity and life cycle with low-temperature energy output capability is still a key issue in current research.

In this work, we report a novel photothermal conversion material by attaching trifluoromethylated azobenzene (Azo_F_) to reduced graphene oxide (rGO). The storage capacity and storage life span as well as the cycling stability performance of Azo_F_-rGO has made great progress. Azo_F_-rGO exhibits great development potential in recyclable and long term photothermal storage.

## 2. Materials and Methods

### 2.1. Materials

3-amino-5-(trifluoromethyl)benzoic acid (99%), 3,5-dimethoxyaniline (99%), sodium nitrite (97%), Na_2_CO_3_ (97%) and NaBH_4_ (97%) were purchased from Aladdin Reagent (Shanghai, China).

### 2.2. Detailed Synthesis Steps

- 3-amino-5-(trifluoromethyl)benzoic acid (1.025 g) was dissolved in the HCl solution (50 mL, 0.5 mol·L^−1^), then NaNO_2_ (0.380 g) was added and reacted at ice bath for 80 min. After dissolving 3,5-dimethoxyaniline (0.765 g) in water, we slowly added the above mixture to it, adjusted the pH to 7 and reacted it in an ice bath for 4 h. Azo_F_ was obtained after further purification (1.255 g, 68%).
- GO was synthesized according to the literature reports [29]. First, we used NaOH (1 mol·L^−1^) solution to change the pH of the GO aqueous solution (300 mL, 0.5 mg·mL^−1^) to 10, then we reacted it at 90 °C for 4 h with NaBH_4_ (180 mg) under N_2_ atmosphere. When the reaction was complete, rGO was obtained by washing the mixture with water multiple times.
- Azo_F_ (0.738 g) was dissolved in the HCl solution (60 mL, 0.5·mol L^−1^), then NaNO_2_ (0.141 g) was slowly added and reacted in an ice bath for 80 min, and the above mixture was slowly added to the rGO solution (62 mL, 1 mg·mL^−1^). The mixture was first reacted at 0 °C for 4 h and then at 30 °C for 16 h. Azo_F_-rGO was obtained by purifying the mixture with water and DMF multiple times.

### 2.3. Characterizations

The FT-IR was gathered from Vertex 70 (Bruker, Karlsruhe, Germany). The XRD was gathered from X‘Pert Pro MPD (PANalytical, Almelo, Holland). Raman spectrum was gathered from LabRAM Aramis (HORIBA, Paris, France). The XPS was gathered from ESCALAB 250Xi (ThermoFisher, Waltham, MA, USA) using C1 s = 284.8 eV for energy calibration procedures, Operation Mode:CAE:Pass Energy 100.0 Ev, software:Thermo Avantage 5.976 and hemispherical energy analyzer were used for the test, the test vacuum was 5 × 10^−9^ Torr, the sample was fixed on the sample stage with conductive glue, the background was buckled through the smart method, and the energy calibration was performed with gold, silver and copper. The TGA was performed on STA449F5 (NETZSCH, Bavaria, Germany). TEM was gathered from Tecnai F20 (FEI, Hillsboro, Oregon, USA). SEM were gathered from SU8010 (Hitachi, Tokyo, Japan). The UV–Vis absorption spectra was performed on SPECORD 50 PLUS (ANALYTIK JENA, Jena, Germany) in the range of 250~550 nm with the resolution of 0.1 nm. The *trans* **→** *cis* transition was introduced by a multiband LED lamp at 365 nm. The *cis* **→** *trans* transition was introduced by a multiband LED lamp at 540 nm. The light intensity was gathered from an optical power meter (PL-MW2000, Bofeilai Technology, Beijing, China). The heat storage density was determined through differential scanning calorimetry (DSC, 214 Polyma, NETZSCH, Bavaria, Germany) under N_2_.

## 3. Results and Discussion

### 3.1. Chemical Structure

**Figure 1:**
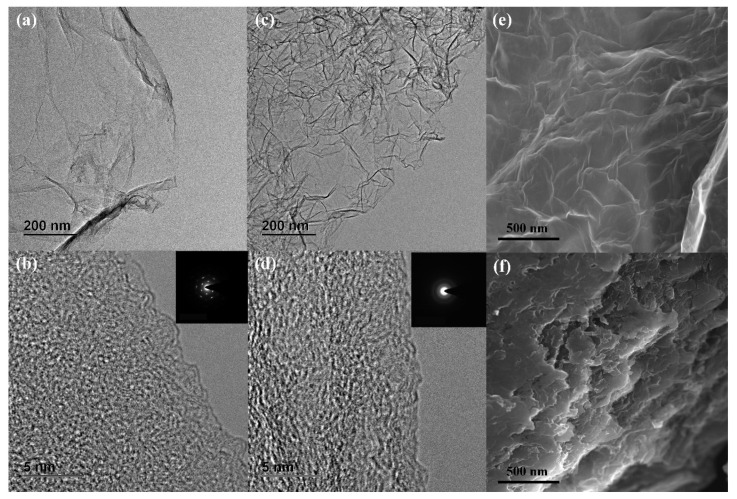
(**a**,**c**) Low resolution TEM images of rGO and Azo_F_–rGO, (**b**,**d**) high resolution TEM images of rGO and Azo_F_-rGO with FFTs, and SEM images of (**e**) rGO and (**f**) Azo_F_–rGO.

As shown in Figure 1a, the low-resolution TEM image of rGO exhibited a smooth structure and its electron diffraction exhibited a hexagonal lattice according to Fast Fourier Transform (FFT) patterns within Figure 1b, demonstrating its good crystallinity. Figure 1c shows that the surface of the material became rough, and the electron diffraction spot of Azo_F_-rGO (Figure 1d) has become a closed loop attributed to the adhesion of Azo_F_ on rGO [30,31]. Furthermore, the SEM of Azo_F_-rGO (Figure 1f) shows a stacking phenomenon compared with rGO (Figure 1e). This phenomenon not only reduced the distance between adjacent graphene layers but also enhanced the intermolecular interaction, resulting in a growth in the storage capacity as well as *τ*_1*/*2_ of Azo_F_-rGO [21]. In addition, it can also be concluded that the distance between layers was reduced based on the XRD results (Figure S2). After the reduction of GO, the (0 0 1) diffraction peak at 11.3° disappeared [32] and was replaced by the (0 0 2) diffraction peak at 22.9° of rGO, and the corresponding grain size was 25.51 nm based on Scherrer formula [33]. After attaching Azo_F_ onto rGO, the 2θ of Azo_F_-rGO has become to 25.2° with the grain size of 22.63 nm, which is consistent with the SEM observation (Figure 1f) [34].

**Figure 2:**
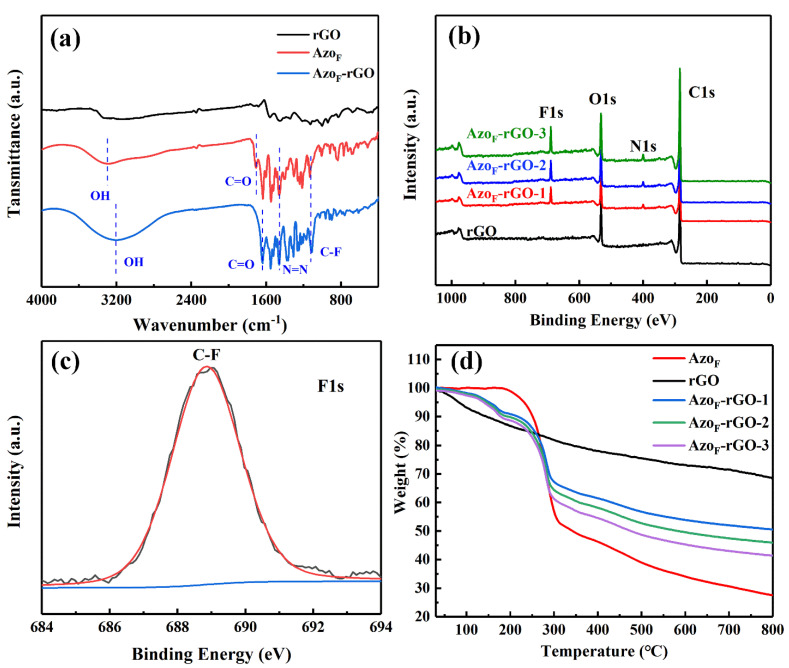
(**a**) FT-IR spectra of rGO, Azo_F_ and Azo_F_-rGO. (**b**) XPS spectra of rGO and Azo_F_-rGO. (**c**) F1s XPS spectra of Azo_F_-rGO. (**d**) TGA spectra of rGO, Azo_F_ and Azo_F_-rGO.

The Azo_F_-rGO had new peaks of –N=N– (1430 cm^−1^) and –CF_3_ (1140 cm^−1^) compared to rGO [35] according to Figure 2a. Moreover, the FT-IR spectra of Azo_F_-rGO and Azo_F_ also showed peaks derived from -OH (3298 cm^−1^) and –C=O (1640 cm^−1^). It can also be seen from Figure 2a that the wavenumbers of -OH and –C=O of Azo_F_-rGO show a significant red shift compared to that of Azo_F_ (3204 cm^−1^ and 1700 cm^−1^), confirming the formation H-bond of Azo_F_ on rGO [36]. XPS results also proven the successful grafting of Azo_F_ on rGO. In addition, the characteristic peaks of Azo_F_ at 287.5 eV and 292.5 eV corresponding to C–N and C–F bond also appeared in Azo_F_-rGO (Figure S3) [35]. Additionally, the fact that there were characteristic peaks of –N=N– (400.3 eV) and –CF_3_ (688.3 eV) in Azo_F_-rGO also confirmed the successful bonding between Azo_F_ and rGO [35].

The high-density adhesion of Azo_F_ onto rGO nanosheets is inextricably linked to the improvement of the performance of Azo_F_-rGO. The decomposition of rGO during the whole heating process was linear according to Figure 2d, and its weight loss mainly attributed to the disappearance of oxygen-containing groups [37]. The Azo_F_ was stable before 185 °C, and its weight loss was attributed to self-decomposition. Additionally, the weight loss of Azo_F_-rGO was caused by the weight loss of Azo_F_ and rGO [27]. Therefore, the attachment density (A_d_) of Azo_F_ on rGO after different time reactions can be obtained based on Equation (1) [38].

(1) $$D_{g}=\frac{R_{p}-R}{R_{p}-R_{a}}\times 100\%$$

where *Ra* is the residual weight percentage of Azo_F_, *R* is the residual weight percentage of Azo_F_-rGO, *Rp* is the residual weight percentage of rGO.

Table 1 shows that the attachment density (A_d_) was 1/40 after the first reaction and increased to 1/16 after the third reaction. The attachment density can also be obtained based on XPS [39]. It can also be seen from Table 1 that the results obtained by XPS and TGA were almost identical. From the above results, it can be concluded that almost every 16 carbon atoms of rGO correspond to one Azo_F_ after the third reaction, which is better than previous research [21,40]. High adhesion density on the one hand helps to form intermolecular hydrogen bonds, while on the other hand it also enhances intermolecular interactions, which improves the storage performance of Azo_F_-rGO [41]. In addition, Raman spectroscopy also proved this result. It can also be seen from Figure S4 that the I_D_/I_G_ value of Azo_F_-rGO-1 (1.14) and Azo_F_-rGO-3 (1.18) was much larger than rGO (1.08), which indicates that the crystal structure of rGO has changed after attachment [31], proving the remarkable attachment density of Azo_F_ on rGO.

### 3.2. Cycling Stability and Storage Performance

**Figure 3:**
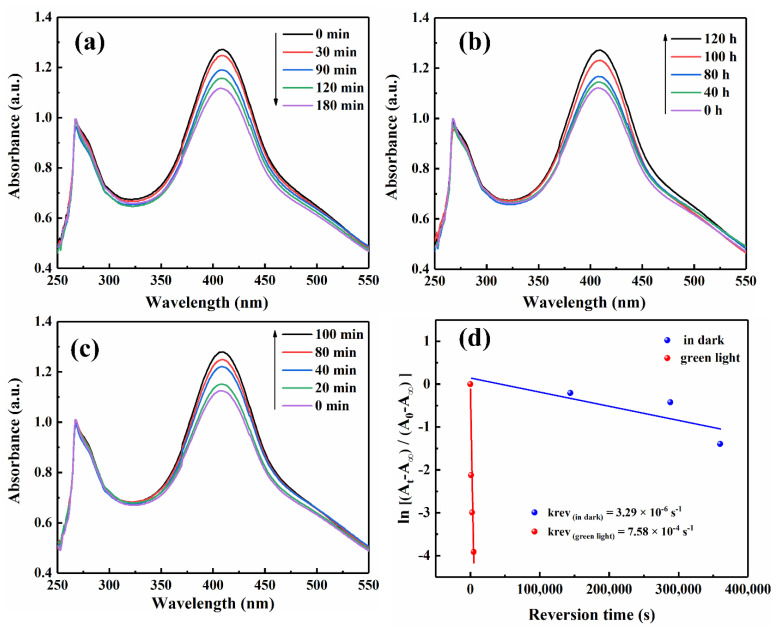
UV–Vis absorption spectra of Azo_F_-rGO-3 (**a**) under UV irradiation, (**b**) in dark conditions, (**c**) under visible light irradiation, (**d**) reversion rates curves of Azo_F_-rGO in different environments.

The optical properties performance of Azo_F_ and Azo_F_-rGO was investigated through time-evolved absorption spectra. It can be seen from Figure 3 that Azo_F_-rGO went through a *trans* **→** *cis* isomerization process under 365 nm ultraviolet light irradiation. Compared with Azo_F_ (*τ*_1*/*2_: 195.2 min), Azo_F_-rGO (*τ*_1*/*2_: 87.7 h) takes more time to complete the isomerization process from *cis*-isomer to *trans*-isomer, indicating that Azo_F_-rGO has better thermal stability than pristine Azo_F_. The same conclusion can be drawn from the fact that the first-order reversion rate constant (*K_rev_*) of Azo_F_-rGO (3.29 × 10^−6^·s^−1^) was much smaller than that of Azo_F_ (1.20 × 10^−4^·s^−1^) under dark conditions derived from Equation (2) [21].

(2) $$\ln\left(\frac{A_{t}-A_{\infty }}{A_{0}-A_{\infty }}\right)=-k_{rev}t$$

where *A*_0_ is the absorption intensity of Azo_F_-rGO and Azo_F_ at metastable state (*cis*-rich) irradiated by UV light, *A_t_* is the absorbance of Azo_F_-rGO and Azo_F_ reversing for “*t*” time and *A_∞_* is the absorption intensity of Azo_F_-rGO and Azo_F_ after complete *cis*-to-*trans* reversion. Moreover, compared to pristine Azo_F_ (Figure S5), Azo_F_-rGO exhibited a lower isomerization degree owing to the intermolecular H-bonds and steric hindrance owing to high attachment density, resulting in a better storage performance of this material. Furthermore, the Δ*Ea* value of the *cis*-isomer of Azo_F_-rGO (1.05 eV) was higher than that of Azo_F_ (0.94 eV) according to Equation (3) [42], which again proves the formation of intermolecular hydrogen bonds [43].

(3) $$E_{a}=-\mathrm{RTln}\frac{hln2}{\tau_{1/2}k_{B}T}$$

where *E_a_* is the activation barrier for *cis*-to-*trans* isomerization process, *T* represents the temperature and ***τ*_1*/*2_** represents the half-life. *k_B_*, *R* and *h* are the Boltzman, universal gas and Plank constants. Additionally, the optical band gap of Azo_F_-rGO complex was estimated to be ~1.8 eV based on the Tauc formula (Figure S6) [44]. The increase in the stability of the *cis*-isomer means extension of the life cycle of Azo_F_-rGO, which is directly related to the large-scale promotion of photoactive chemical heat storage materials.

Similar to the length of the life cycle, whether the controllable heat release under external stimuli can be achieved is critical to the future application value of Azo_F_-rGO. Figure 3c showed that compared with dark conditions, the irradiation of green light (540 nm) significantly accelerated the recovery process of Azo_F_-rGO from *cis* -isomer to *trans*-isomer. Compared with dark conditions, the result that *K_rev_* (7.58 × 10^−4^·s^−1^) was significantly larger under green light irradiation also confirmed the conclusion of faster reversion. The same effect can also be achieved by absorbing heat from the external environment according to DSC. The reason for this phenomenon is that the cis-isomer can absorb energy from external stimuli to overcome the energy barrier of configuration reversion isomerization, thereby achieving the purpose of accelerating energy output [45,46]. The above results show that Azo_F_-rGO has successfully possessed the controllable heat output capability.

**Figure 4:**
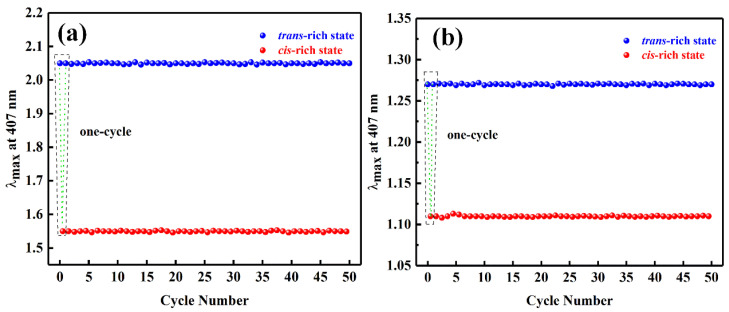
Stability performance of (**a**) Azo_F_ and (**b**) Azo_F_–rGO-3 for 50 times.

The stability of repeated *cis ↔ trans* configuration transformations of Azo_F_-rGO and Azo_F_ has also been studied. It can be seen from Figure 4 that both Azo_F_-rGO and Azo_F_ have no significant decrease in the absorption intensity at 407 nm after repeated irradiation of ultraviolet light (365 nm) and visible light (540 nm) for 50 times, which shows that they have outstanding isomerization stability. The Azo_F_-rGO can not only be stored for a long time under the premise of ensuring the storage effect, but also can control the output of the stored energy, which is essential for actual photothermal conversion.

**Figure 5:**
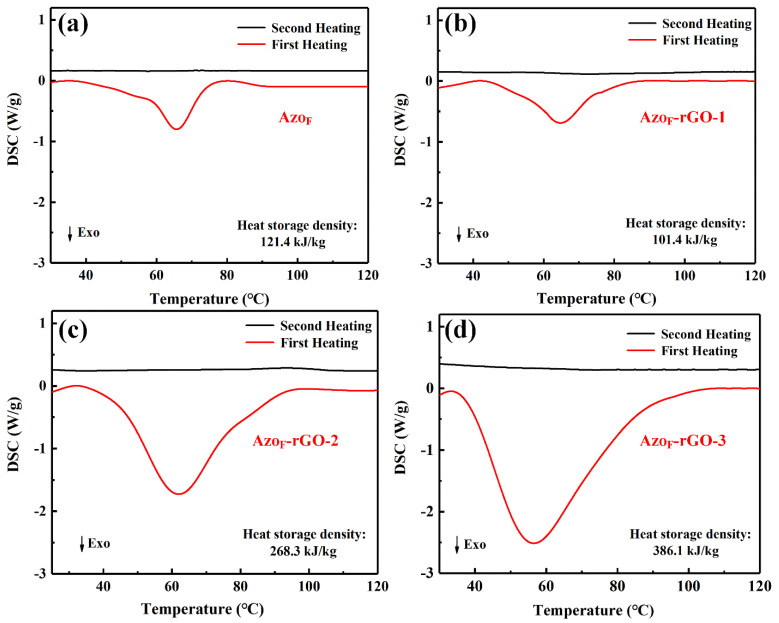
DSC (differential scanning calorimetry) traces of (**a**) Azo_F_ and (**b**–**d**) Azo_F_-rGO after 1, 2 and 3-times reaction.

The photothermal storage capacity of Azo_F_ and Azo_F_-rGO was investigated through DSC [7]. All objects were stable between 10–140 °C based on TGA. Azo_F_ and Azo_F_-rGO released significant heat under the first round of heating stimulation, but no heat was released during the second round according to Figure 5. The above results prove that the research subjects have released all the energy stored through the configuration transformation in the form of heat. Furthermore, most photothermal storage materials start to release the stored energy after 100 °C, while this kind of heat storage material can start energy output at 35 °C, which is a milestone in achieving fast energy output at lower temperatures [7].

It can be seen from Figure 5 that the heat storage density of Azo_F_-rGO-3 has reached to 386.1 kJ kg^−1^, which shows a significant increase over Azo_F_ (121.4 kJ kg^−1^). This is because of the close-packed orderly distribute of Azo_F_ on rGO as a result of high attachment density, which strengthens the intermolecular interaction [23]. In addition, high attachment density also enhances the steric hindrance and promotes the formation of H-bonds, which further increases the photothermal storage capacity [47]. The reason for Azo_F_-rGO-1 showing less effectiveness compared to the Azo_F_ is the low attachment density, which leads to weak intermolecular interaction and therefore relatively low energy density. Moreover, the heat storage density of Azo_F_-rGO-3 was also higher than Azo_F_-rGO-1 and Azo_F_-rGO-2, which shows that the attachment density was positively correlated with great storage performance.

**Figure 6:**
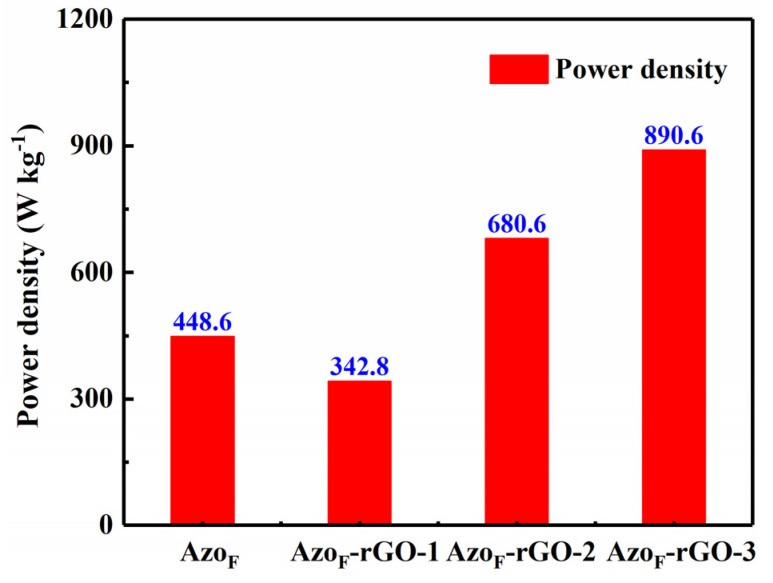
Power density of Azo_F_ and Azo_F_-rGO after 1, 2 and 3-times reaction.

Similar to heat storage density, power density is also a key element to measure the possibility of practical application of Azo_F_-rGO. It can be seen from Figure 6 that the power density of Azo_F_-rGO-3 was 890.6 W kg^−1^, which shows a huge improvement compared to Azo_F_ (448.6 W kg^−1^). Furthermore, the power density of Azo_F_-rGO-3 was also higher than Azo_F_-rGO-1 and Azo_F_-rGO-2, which shows that the attachment density is directly related to the heat output performance. It is worth noting that high power density means fast output of energy, which further increases the feasibility of practical application of Azo_F_-rGO. As shown in Table 2, the performance of Azo_F_-rGO in many aspects has been greatly improved compared to other similar materials [7,15,48,49]. The above results demonstrate that Azo_F_-rGO, which not only exhibits remarkable photothermal capacity but also equipped with low temperature energy output capability, has shown great development potential in achieving the goal of efficient photothermal storage.

## 4. Conclusions

In summary, Azo_F_-rGO with good photothermal storage performance, outstanding storage lifespan and low-temperature energy output capability has been proven to be a great photothermal conversion material. The formation of hydrogen bonds and the enhancement of intermolecular interactions owing to the high attachment density has simultaneously achieved the improvement of the heat storage density (max. 386.1 kJ kg^−1^), power density (max. 890.6 W kg^−1^) and half-life (up to 87.7 h) of Azo_F_-rGO for photothermal storage. Azo_F_-rGO also exhibits exceptional cycling stability, which realizes long-term recyclability and efficient and pollution-free utilization of solar energy in a closed system. Furthermore, Azo_F_-rGO can start energy output at 35 °C, which shows that the goal of low-temperature energy output has been achieved. The above results indicate that Azo_F_-rGO, combining outstanding photothermal capacity with a long-life cycle as well as low-temperature energy output capability, is a prominent photothermal conversion material with great practical application value.

## Figures and Tables

**Table 1:** A_d_ of Azo_F_ on rGO.

	TGA		XPS			
Reaction Times	*D_g_* (%) ^a^	A_d_	Element Content (%)			A_d_
			C	F	O	
Azo_F_-rGO-1	43.41	1:40.1	77.42	4.13	15.71	1:40.2
Azo_F_-rGO-2	52.95	1:27.3	74.13	5.09	17.39	1:27.7
Azo_F_-rGO-3	65.73	1:16.0	71.07	6.64	17.90	1:16.1

^a^*Dg* is the average weight percentage of Azo_F_ in Azo_F_-rGO at 600 °C, 700 °C and 800 °C.

**Table 2:** Performance of different photothermal conversion materials.

Photothermal Conversion Material	Energy Density (kJ mol^−1^)	Power Density (W mol^−1^)	Half-Life (h)	Ref.
Azo-diacetylene polymer	176.2	1289.5	27.8	[48]
Azo-SWCNT complex	92.0	457.1	0.5	[7]
Azo-PCM complex	79.3	–	–	[15]
Azo-alkyl polymer	89.0	148.6	55	[49]
Azo_F_-rGO-3 complex	367.7	848.6	87.7	This paper

## Data Availability

The data presented in this study are available in [insert article or Supplementary Material].

## Supplementary Materials

The following are available online at https://www.mdpi.com/1996-1944/14/6/1434/s1, Figure S1: ^1^H NMR, ^13^C NMR and ^19^F NMR spectra of Azo_F_, Figure S2: XRD patterns of GO, rGO, Azo_F_-rGO, Figure S3: C1s region in XPS spectra of (a) rGO, (b) Azo_F_-rGO and (c) N1s region in XPS spectra, Figure S4: Raman spectra, Figure S5: Time-evolved absorption spectra of Azo_F_ and Figure S6: UV-Vis absorption spectra of Azo_F_-rGO powder at room temperature (25 °C).
