# Supplementary material for: A Low-Temperature Heat Output Photoactive Material-Based High-Performance Thermal Energy Storage Closed System
Source: Materials (Basel). 2021 Mar 16;14(6):1434. doi: 10.3390/ma14061434 (PMC8000957; doi:10.3390/ma14061434)
Supplement: Supplementary file 1 [file materials-14-01434-s001.pdf]

materials

MDPI

Article

A Low-Temperature Heat Output Photoactive Material-Based High-Performance Thermal Energy Storage Closed System

Xiangyu Yang [1,2], Shijie Li [2], Jin Zhang [2], Xiaomin Wang [1], Yongzhen Wang [1,*] and Jianguo Zhao [1,2,*]

[1] College of Materials Science and Engineering, Taiyuan University of Technology, Yingze West Street, Taiyuan 030024, China; yangxiangyu0039@link.tyut.edu.cn (X.Y.); wangxm62@126.com (X.W.)

[2] Institute of Carbon Materials Science, Shanxi Datong University, Xingyun Street, Datong 037009, China; li841974@sina.com (S.L.); zhangjin50@hrbeu.edu.cn (J.Z.)

* Correspondence: wangyz62@163.com (Y.W.); zhaojianguo@sxdtdx.edu.cn (J.Z.)

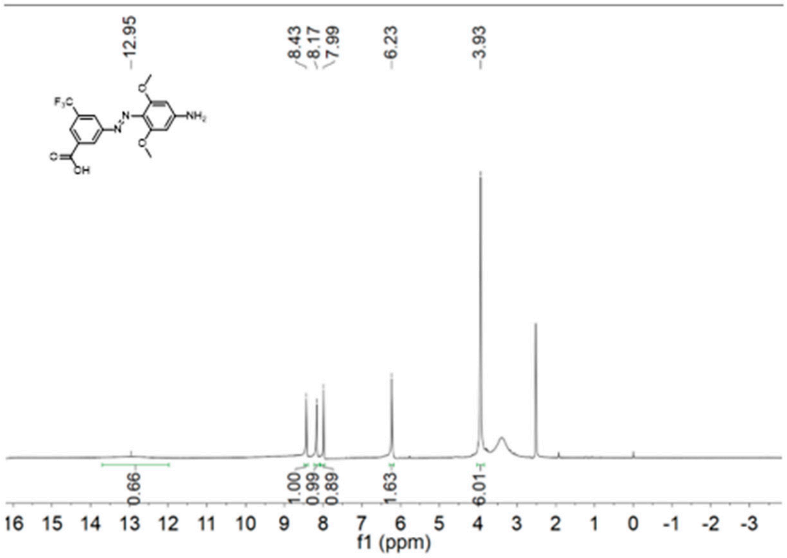

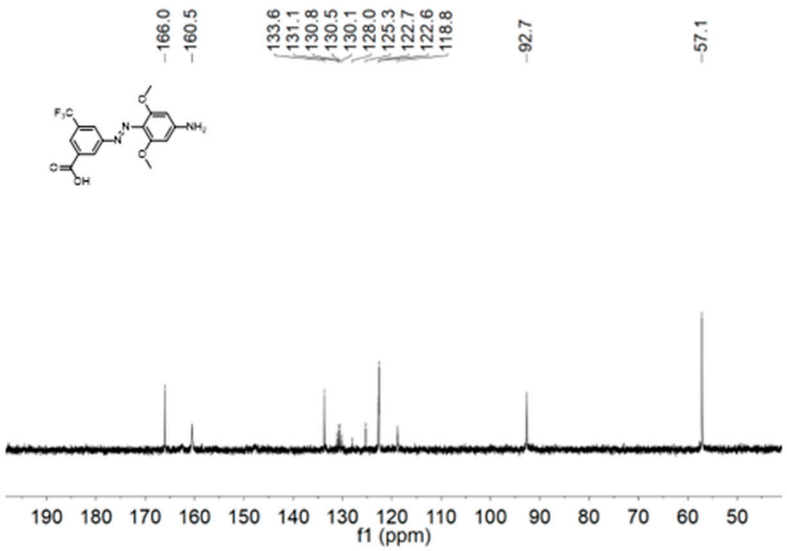

Citation: Yang, X.; Li, S.; Zhang, J.; Wang, X.; Wang, Y.; Zhao, J. A Low-Temperature Heat Output Photoactive Material-Based High-Performance Thermal Energy Storage Closed System. *Materials* **2021**, *14*, 1434. https://doi.org/10.3390/ma14061434

Academic Editor: Mariano Palomba

Received: 27 December 2020
Accepted: 25 February 2021
Published: 16 March 2021

Publisher's Note: MDPI stays neutral with regard to jurisdictional claims in published maps and institutional affiliations.

Copyright: © 2021 by the authors. Licensee MDPI, Basel, Switzerland. This article is an open access article distributed under the terms and conditions of the Creative Commons Attribution (CC BY) license (http://creativecommons.org/licenses/by/4.0/).

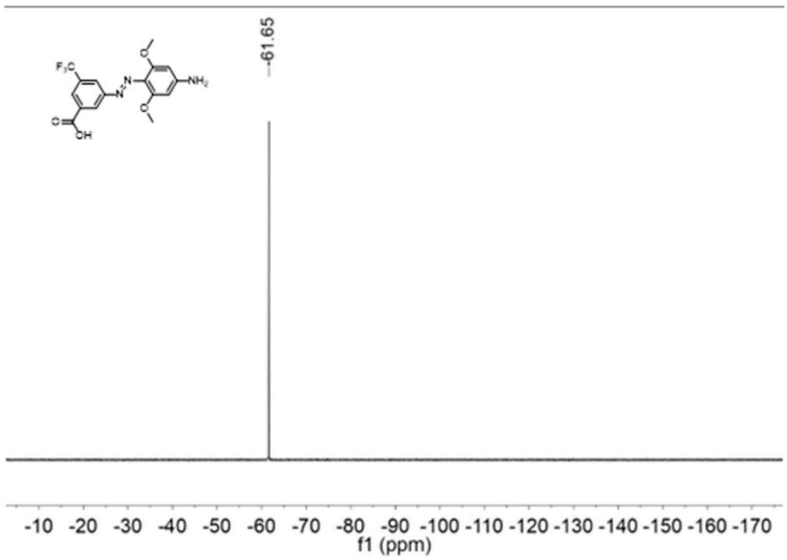

Figure S1. ^{1}H NMR, ^{13}C NMR and ^{19}F NMR spectra of Azo_F.

The Nuclear Magnetic Resonance (NMR) spectra was measured on a AV-400 spectrometer (Bruker, Germany) with trimethylsilyl and CCl_3F as the internal reference. High-resolution mass spectrometry (HRMS) was recorded on an Q-TOF6540 LC/MS Analyzer (Agilent, USA). And the Characterization data of Azo_F: ^{1}H NMR (400 MHz, DMSO-d_6) δ 12.95 (s, 1H), 8.43 (s, 1H), 8.17 (s, 1H), 7.99 (s, 1H), 6.23 (s, 2H), 3.93 (s, 6H); ^{13}C NMR (101 MHz, DMSO-d_6) δ 166.0, 160.5, 133.6, 130.6 (q, *J* = 32.2 Hz), 128.0, 125.3, 122.7, 122.6, 118.8, 92.7, 57.1; ^{19}F NMR (376 MHz, DMSO-d_6) δ -61.65. HRMS (ESI) m/z calcd. for $C_{16}H_{14}F_3N_3O_4$ $[M+H]^+$ 368.0936, found 368.0933.

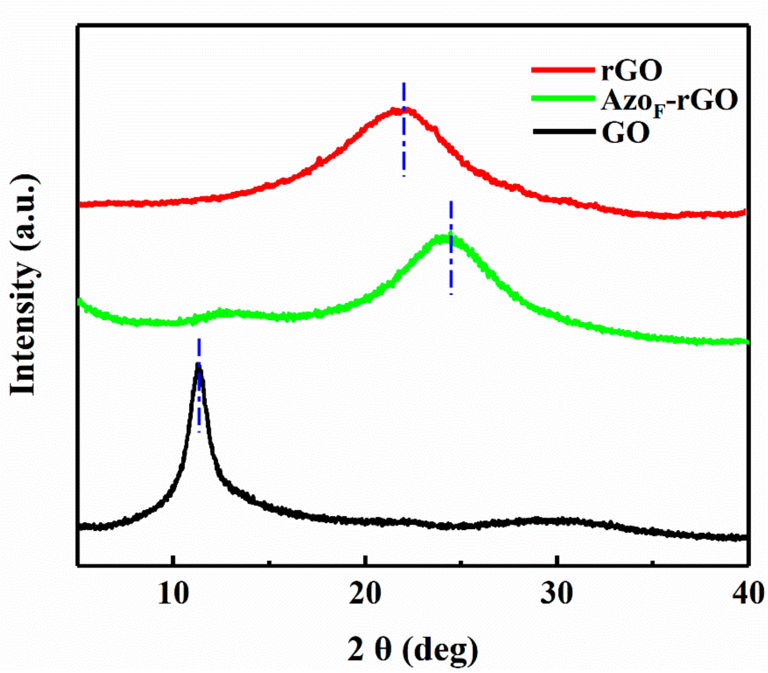

Figure S2. XRD patterns of GO, rGO, Azo_F-rGO.

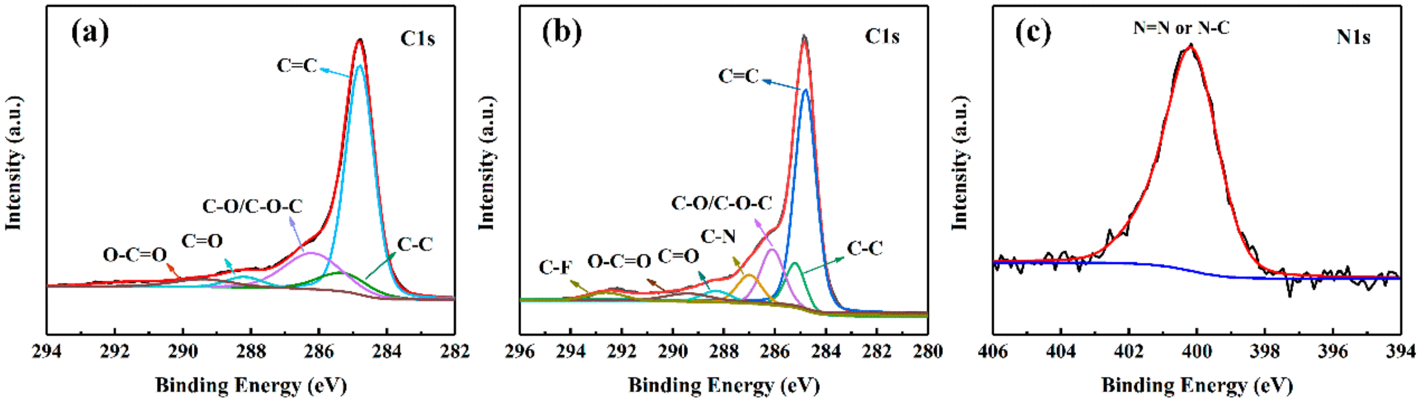

Figure S3. C1s region in XPS spectra of (**a**) rGO, (**b**) AzoF-rGO-3 and (**c**) N1s region in XPS spectra of AzoF-rGO.

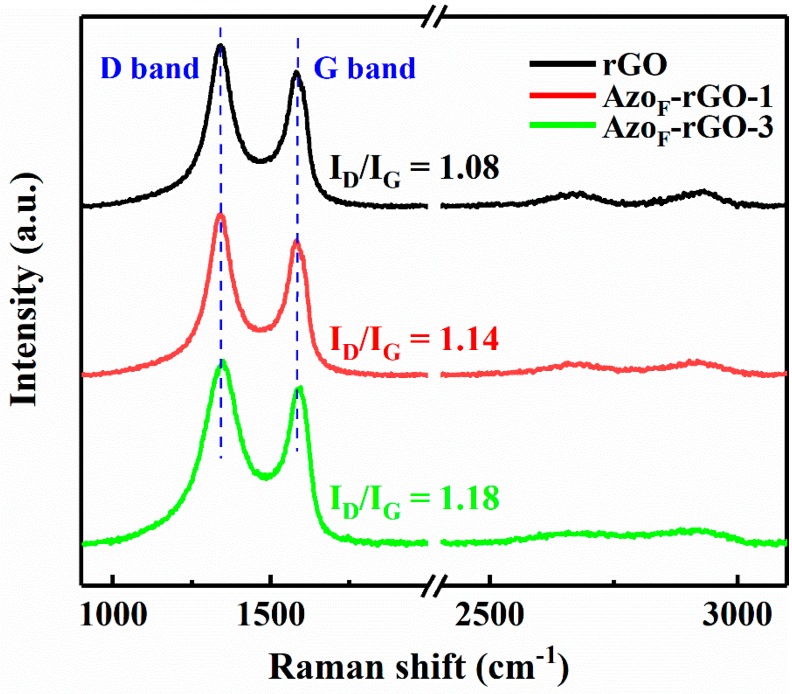

Figure S4. Raman spectra of rGO and AzoF-rGO with different grafting density. It show black curve for rGO, red curve for low attachment density AzoF-rGO and green curve for high attachment density AzoF-rGO, respectively.

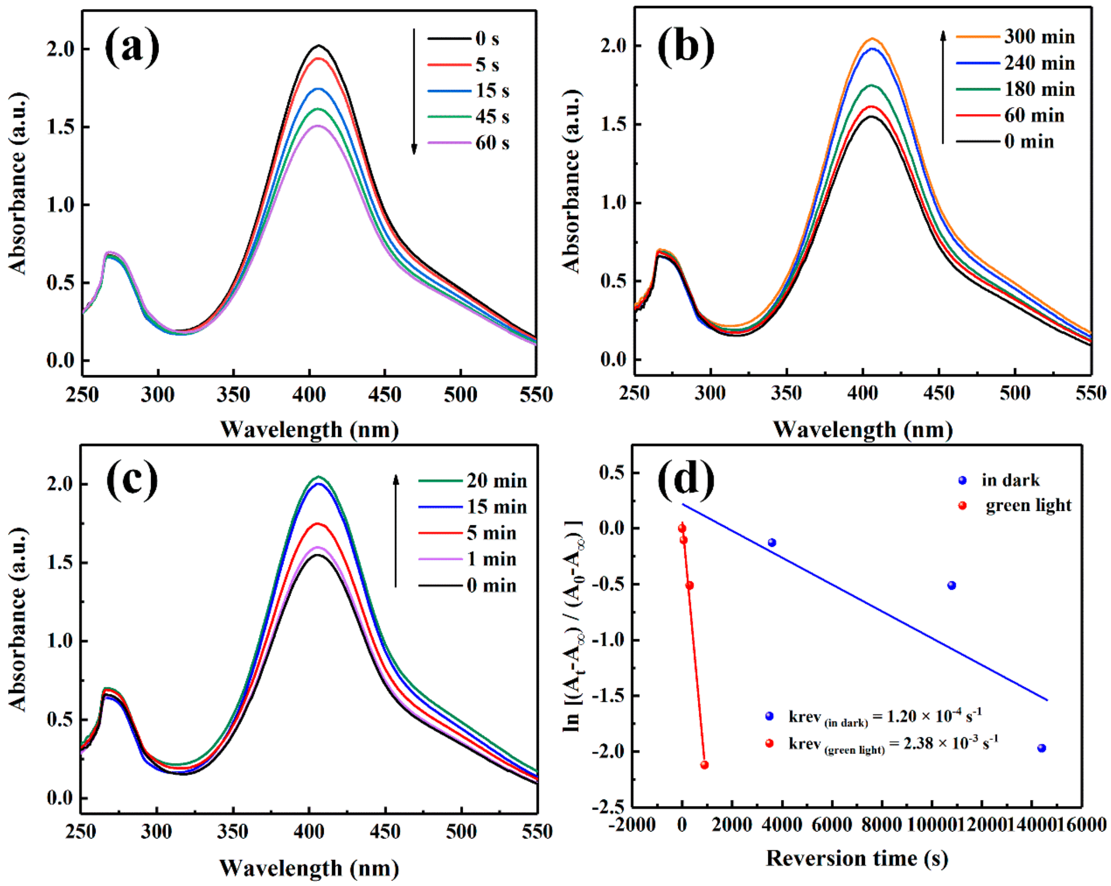

Figure S5. Time-evolved absorption spectra of Azof at room temperature (25 °C). (**a**) Irradiated with UV light at 365 nm, (**b**) kept in the dark and (**c**) irradiated by visible light at 520 nm. (**d**) First plots of *cis*-to-*trans* reversion of AzoF with different krev (the inset).

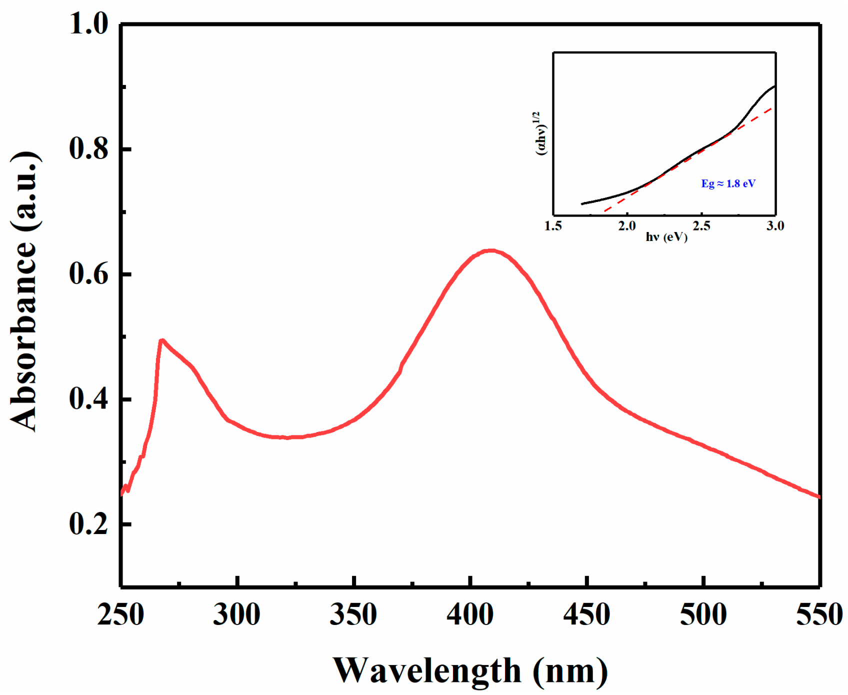

Figure S6. UV-Vis absorption spectra of AzoF-rGO powder at room temperature (25 °C). The inset shows the extracted Tauc plot.
